# Tactile stimulus discrimination in adolescents with anorexia nervosa: a behavioral and neurophysiological study

**DOI:** 10.1038/s41398-026-04264-3

**Published:** 2026-07-23

**Authors:** Jule Romea Lammers, Hugo Romero Frausto, Anke Dalhoff, Selina Hansen, Kati Roesmann, Markus Junghöfer, Ida Wessing

**Affiliations:** 1 Department of Child and Adolescent Psychiatry, University Hospital Münster, Münster, Germany; 2 Department of Psychosomatic Medicine and Psychotherapy, University Hospital Münster, Münster, Germany; 3 Institute of Psychology, Clinical Psychology and Psychotherapy in Childhood and Adolescence, University of Osnabrück, Osnabrück, Germany; 4 Institute for Biomagnetism and Biosignalanalysis, University of Münster, Münster, Germany; 5 Otto Creutzfeldt Center for Cognitive and Behavioral Neuroscience, University of Münster, Münster, Germany

**Keywords:** Psychiatric disorders, Neuroscience

## Abstract

Body image disturbance (BID) is a key symptom of anorexia nervosa (AN) and involves body size overestimation. Although this overestimation might be related to perceptual deficits, little is known about tactile perception in AN. To clarify the role of putative tactile deficits in BID, the present study investigated 36 adolescent female AN patients and 41 matched healthy controls (HC) using a tactile oddball paradigm during parallel EEG and MEG (EMEG) measurement. Tactile perception was behaviorally tested via the Touch Test (tactile perception threshold), a deviant count task, and a tactile stimulus discrimination task. Compared to HC participants, AN patients had similar tactile perception thresholds, but performance in the deviant count task and the tactile stimulus discrimination task was poor. Non-parametric cluster permutation tests on estimated neural source activity of evoked EMEG responses revealed that AN patients and HC did not differ regarding the neural differentiation between deviant and standard stimuli (oddball effect). However, estimated neural activity was globally reduced in the inferior temporal cortex and (by trend) in the posterior parietal cortex. These findings argue against the idea that behavioral discrimination deficits of tactile stimuli in AN are grounded in neural stimulus discrimination deficits. Instead, the overall reduced neural activity in the inferior temporal and posterior parietal cortex might reflect aberrant integration of neural tactile representations into a coherent multisensory body representation.

## Introduction

Anorexia nervosa (AN) is an eating disorder often beginning in adolescence and is characterized by significantly low body weight, restriction of food intake, and body image disturbance (BID). Considering high mortality rates [1] as well as high relapse rates [2, 3], there is an urgent need for more effective and personalized treatment options for AN [4]. The severity or persistence of BID has been linked to treatment outcomes [5, 6], relapses [7], and long-term outcomes [8]. Thus, for the development of mechanism-based treatment approaches, a better understanding of BID seems promising.

The term ‘body image’ is a multidimensional construct consisting of cognitive-affective and perceptive components [9]. Typically, AN patients show impairments of both, the cognitive-affective component, i.e. negative attitudes, thoughts and feelings regarding the body, and also the perceptive component, which is typically inferred from body size overestimation (e. g., [10]). There is evidence that a greater overestimation of one’s body size in adolescent AN patients is associated with a worse long-term course of the disease [8] and that overestimation is reduced but persists even after successful weight restoration [11, 12]. Despite its high clinical relevance, only a few treatment approaches directly targeted body size overestimation [13] or non-visual perceptive aspects of BID [14]. The background of the perceptive component of BID is still poorly understood and often controversially discussed [15]. It is unclear whether body size overestimation is mainly driven by cognitive-affective aspects such as negative thoughts and feelings, and/or if altered perceptual processes are key [15].

Some researchers suggest that body size overestimation in AN is primarily driven by body dissatisfaction and the pursuit of thinness [16–18]. Indeed, body size overestimation correlates with body dissatisfaction [16, 18], and no such overestimation occurs when evaluating other people’s bodies [19]. However, even emotionally neutral body parts, such as the hands, are overestimated more by AN patients than HC [20].

Neuroimaging studies provide further insights into these distortions. The posterior parietal cortex (PPC), crucial for integrating multisensory input into coherent body representations, appears impaired in AN, with deficits extending to both body-related and unrelated stimuli [21, 22]. fMRI research shows altered activity in the PPC when patients view body-related stimuli, consistent with structural MRI findings of reduced gray matter in this region [23]. Consistently, a recent meta-analysis found hyperactivity in the right temporoparietal lobe in response to visual body cues in AN patients [24]. Resting-state fMRI findings showed reduced connectivity between sensorimotor and visual networks [25]. These findings suggest that disrupted multisensory integration, centered in the PPC, might underly perceptive BID in AN [24].

In the past, the primary research focus regarding possible perceptual impairments has been the visual modality [26]. To date, only a few studies have shed light on tactile perception in AN patients. The few existing behavioral studies on tactile perception in AN have used tasks of varying complexity (see [27] and, [26] for a review). Perceptual thresholds of pressure from a single stimulus applied to the skin were not significantly different from HC [28] or were reduced on the abdomen, i.e., in this region tactile stimuli were perceived better by AN patients [29]. Furthermore, AN patients did not differ from HC in identifying the location of tactile stimuli on their back or abdomen [30] or on one of their fingers [31] and also had no difficulty distinguishing which of two tactile stimuli had a longer duration [28].

However, several studies reported deficits in AN patients: Epstein et al. [31] found that AN patients had difficulties identifying which two fingers were touched simultaneously when their eyes were closed. Keizer et al. [29] and Spitoni et al. [28] observed that in a two-point discrimination task, AN patients’ threshold was higher on both the arm and abdomen. Thus, compared to HC, AN patients required a greater distance between two stimuli to perceive them as separate. Moreover, in a tactile estimation task, participants were asked to estimate the distance between two tactile stimuli on the skin. This task requires recourse to implicit body representations [32]. AN patients overestimated the distance of tactile stimuli on both the abdomen and arm, suggesting that the implicit representation of their body shape is larger than their actual body shape [13, 28, 29, 33]. Spitoni et al. [28] found that this difference was more pronounced when stimulation occurred on the horizontal axis, suggesting that AN patients specifically perceive their bodies to be wider. This difference appears to persist after treatment unless specific training is applied [13]. However, two studies have been unable to replicate performance deficits in the tactile estimation task in AN [12, 34].

The behavioral findings regarding tactile perception in AN thus show a heterogeneous picture. The tactile perception threshold seems to be unaffected in AN. However, in some of the studies, disturbances were observed regarding tactile stimulus differentiation and regarding tactile tasks that require access to implicit body representations. Behavioral tactile perception deficits were not correlated with BMI in previous studies [29, 33, 35] and persisted after weight gain [13]. This suggests that these deficits most likely reflect a persistent trait [27] that is not elicited solely by the underweight condition. These findings are congruent with the observation that body size overestimation often persists after weight restoration [11, 12].

To date, to our knowledge, no published study has investigated tactile perception in AN patients using neurophysiological methods. We aimed to elucidate the neural mechanisms of tactile processing in AN by using simultaneous electro- and magnetoencephalography (EMEG), as these methods provide complementary spatial localization, and their high temporal resolution supports differentiating earlier sensory processes from later more integrative processes in time. During EMEG recording, adolescent AN patients and age-, gender-, and IQ-matched healthy controls (HC) were presented with a tactile oddball paradigm, and tactile perception was tested via a deviant count task and a tactile stimulus discrimination task. On the behavioral level, tactile perception deficits in AN patients should be indicated by worse task performance. On the neural level, we considered two possible outcomes: Reduced neural stimulus discrimination in early time windows and primary somatosensory areas would suggest fundamental tactile perception deficits in AN. In contrast, reduced activity in later time windows and the PPC would indicate sensory integration deficits in AN.

## Methods

### Recruitment

This study included female patients with AN aged 12–19 years and HC participants matched for gender, age, and IQ (see Behavioral Tests). General exclusion criteria were intellectual disability, suicidality, substance abuse, and EMEG-related exclusion criteria. AN patients were recruited during inpatient treatment on a specialized ward for eating disorders at the Department of Child and Adolescent Psychiatry, University Hospital Muenster, Germany. All diagnoses were given by the treating clinician and then additionally confirmed by structured clinical interviews (see Clinical Characterization). We included mostly AN patients with restrictive type (Table 1) and excluded AN patients with comorbid pervasive developmental disorders or psychotic disorders.

**Table 1:** Sample characteristics.

Group comparison	AN	n	HC	n	Statistics	*p (two-sided)*	*d*	
Age	15.76 ± 1.64	36	16.40 ± 1.66	41	*t*(75) = 1.689	*p* = 0.095*	*d* = 0.39	n.s.
IQ	106.03 ± 13.18	36	106.29 ± 13.76	41	*t*(75) = 0.086	*p* = 0.932	*d* = 0.02	n.s.
Handedness (right/left/both)	31 / 1 / 1	33	37 / 3 / 1	41	*χ²*(2, 74) = 0.672	*p* = *0.714*		n. s.
BMI	15.61 ± 1.35	36	20.44 ± 2.03	41	*t*(75) = 12.151	*p* < 0.001	*d* = 2.78	AN < HC
BMI-SDS	−2.42 ± 1.01	36	−0.12 ± 0.64	41	*t*(57.825) = 11.751	*p* < 0.001	*d* = 2.76	AN < HC
EDE-I (ED Interview)	3.48 ± 1.35	36	0.19 ± 0.21	41	*t*(36.460) = 14.516	*p* < 0.001	*d* = 3.53	AN>HC
EDI-C (ED Questionnaire)	221.58 ± 54.61	36	102.51 ± 34.70	41	*t*(75) = 11.560	*p* < 0.001	*d* = 2.64	AN>HC
BSQ (Body Dissatisfaction)	119.00 ± 38.21	36	54.38 ±17.93	40	*t*(48.540)= 9.271	*p* < 0.001	*d* = 2.20	AN>HC
BDI-II (Depression)	26.28 ± 11.46	36	3.76 ± 4.64	41	*t*(44.988) = 11.064	*p* < 0.001	*d* = 2.64	AN>HC
SCARED (Anxiety)	29.63 ± 13.66	35	14.32 ± 7.76	41	*t*(74) = 6.120	*p* < 0.001	*d* = 1.41	AN>HC
**AN group clinical details**								
AN Subtype			*n* = 36		Restricting type			*n* = 31
					Binge-eating/purging type			*n* = 4
					Atypical AN			*n* = 1
In-patient treatment duration before study participation			31.72 ± 33.38 days					
First in-patient treatment			*n* = 32		Repeated in-patient treatment			*n* = 4
Psychotropic medication			*n* = 6		Olanzapine**			*n* = 2
					Fluoxetine			*n* = 2
					Citalopram			*n* = 1
					Melatonin			*n* = 1
					Promethazine**			*n* = 1
Patients with comorbid disorders			*n* = 13		Depression			*n* = 10
					Anxiety disorder			*n* = 2
					OCD			*n* = 1
					PTBS			*n* = 1

*Despite a trend toward higher age in the HC group, ANCOVA analyses confirmed that all reported group effects and interactions remained robust when controlling for age. ** To exclude potential effects of sedating medication, all relevant analyses were repeated excluding the two patients taking such medication, and the results remained unchanged.

*BMI* body mass index, *BMI-SDS* body mass index standard deviation score, *EDE-I* eating disorder examination interview, *ED* eating disorder, *EDI-C* eating disorder inventory for children, *BSQ* body shape questionnaire, *BDI-II* beck depression inventory–II, *SCARED* screen for child anxiety related emotional disorders.

HC participants were recruited from secondary schools in Muenster. HC participants completed the same structured diagnostic procedures as AN patients. Exclusion criteria for HC were clinically relevant psychopathology (telephone screening, structured clinical interviews, questionnaires) and over- or underweight (BMI age percentile >90 or < 10).

All participants and their parents were informed of the study protocol in oral and written form and gave written informed consent. Participants received an expense allowance of 10 Euros per hour for their participation. The study was approved by the ethics committee of the Medical Association of Westphalia-Lippe, in accordance with the Declaration of Helsinki (2016-457-f-S).

### Clinical characterization

AN diagnoses were confirmed by the Eating Disorder Examination Interview (EDE-I; [36]). Comorbid diagnoses were confirmed using the Diagnostic Interview for Mental Disorders in Children and Adolescents [37]. Participants filled in German versions of five self-report questionnaires. Psychopathology and body image were assessed using the Eating Disorder Inventory for Children (EDI-C; [38]), Body Shape Questionnaire (BSQ; [39]), Beck’s Depression Inventory (BDI-II; [40]) and Screen for Child Anxiety-Related Emotional Disorders (SCARED-D; [41]). Participants’ handedness was assessed using the Fazio Laterality Inventory (FLI; [42]).

### Behavioral tests

Intelligence was tested using a computerized language-free IQ test (CFT-20R; Basic Intelligence Test Scale 2; [43]).

The tactile perception threshold at the right index finger was determined using the Touch Test TM Sensory Evaluator (North Coast Medical, Inc., Morgan Hill, CA, USA; details in Supplement 1).

Perceptive BID was measured using a Body Size Estimation (BSE) task [44]. In this task, participants estimate the circumference of their waist, upper arm, and thigh by employing a rope. Afterwards, the length of the rope is measured using a measuring tape. Finally, the real circumference of each body part is determined using the same rope and measuring tape. The estimated and measured circumferences of each body part are then used to calculate body perception indices (BPI=estimated/measured×100), and their mean determines the total BPI.

### Experimental data

#### Procedure

Participants were introduced to the measurement chamber and the paradigms. Digital head renderings were recorded using a 3D tracking device (Polhemus, Colchester, USA). Participants wore an electrode skullcap for EEG measurements and were placed in a supine position in the MEG scanner (see EMEG Data Acquisition and Analysis). During the recording, participants were asked to leave their eyes open. The EMEG recording session included the tactile oddball paradigm reported here as well as the visual body size estimation task, reported by Romero et al. [45].

#### Tactile oddball paradigm

**Fig. 1:**
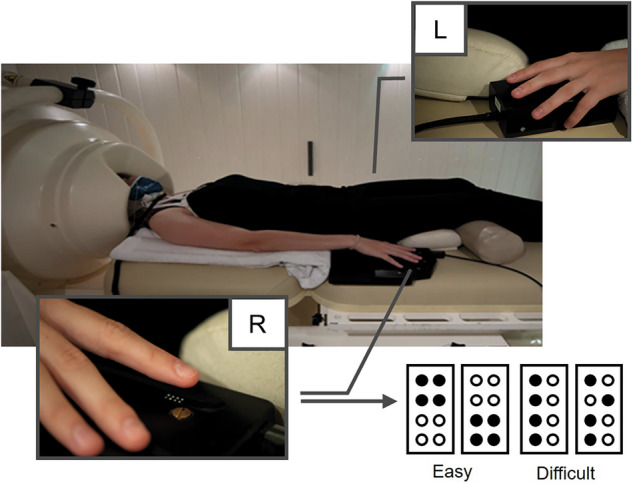
Supine position of patients and controls during MEG/EEG data recording. Bottom left: Braille stimulation device used to stimulate the index finger of the right hand during the tactile oddball paradigm and the tactile stimulus discrimination task. Top right: Responds for the tactile stimulus discrimination task were given with the left hand using a button box. Bottom right: Tactile patterns in the easy and difficult subtasks.

During the tactile oddball task (Fig. 1) the distal phalanx of the participant’s right index finger was placed on a tactile stimulator (Braille stimulator). The Braille stimulator (Metec AG, Stuttgart, Germany) contains eight individually piezo-controllable plastic pins grouped in a 2 × 4 arrangement. The distance of 2.5 mm between adjacent pins is close to the maximum tactile acuity of the fingertip [46]. Participants were presented with different tactile patterns (standard, deviant) in two subtasks (easy, difficult). In the easy subtask, four pins were raised in either the upper or lower section of the array (standard vs. deviant). In the difficult subtask, pins were aligned in the left column (standard), with deviants involving a one-pin shift to the right column (Fig. 1). Each tactile pattern was presented for 500 ms, with a jittered inter-stimulus-interval of 500 ms +/- 100 ms (∼390 ± 10 trials). In about 82% of the trials (∼320 ± 10 trials) the standard appeared, and in about 18% (70 trials) the deviant appeared in a pseudo-randomised order (maximum of three repetitions of the same tactile pattern, equal transition probability). The order of the subtasks (easy, difficult) was randomized. Participants had to silently count the number of deviants, focussing attention on the stimuli. Performance in this task was defined as the absolute value of the number of deviants counted minus the actual number of deviants (*N* = 70) and is called *deviant count*. Differences in each direction (participant counted more or less deviants than were actually presented) were equally considered. The maximum absolute deviant count was set to 70, assuming that both counting no deviant or more than double the deviants equally reflects a failure to complete the task.

#### Tactile stimulus discrimination

The ability to discriminate the tactile patterns presented in the tactile oddball paradigm was additionally tested in a tactile stimulus discrimination task. There were again two subtasks (easy, difficult) and, as this task was rather challenging, the easy subtask was always performed first. In each subtask, 50 trials (25 trials per pattern, 500 ms each) were presented in a pseudo-randomised order. Participants identified each pattern by pressing one of two different buttons with the left index and middle fingers. The next pattern followed immediately after a button press or if no response was detected after 1.5 s.

#### EMEG data acquisition and analysis

Tactile evoked magnetic fields were recorded using a 275-channel whole-head sensor system MEG with first-order axial SQUID gradiometers (Omega 275, CTF MEGTM, VSM Medtech Ltd., Coquitlam, British Columbia, Canada). In parallel, tactile evoked potentials were recorded using either a 57-channel (34 HC, 18 AN) or an 80-channel (20 HC, 20 AN) EEG skullcap (Easycap GmbH, Germany), according to the International 10–20 system [47]. During EEG recording, FCz was used as an online reference point and re-referenced offline to an average reference. Head position and head movements were tracked using landmark coils in each ear channel and on the nasion. Continuous EMEG data were recorded from 0–150 Hz using a sample rate of 600 Hz and then down sampled to 300 Hz. A zero-phase Butterworth third-order high-pass filter [12 dB/oct] and a zero-phase fourth-order low-pass filter [24 dB/oct]) with cut-off frequencies of 0.01 Hz and 48 Hz, respectively, were applied. Single-trial data editing and artefact rejection were conducted using the method for statistical control of artefacts in high-density EMEG data [48]. Single epochs of 800 ms (200 ms before to 600 ms after stimulus onset) were averaged corresponding to experimental conditions. A pre-stimulus interval of 150 ms was used for baseline adjustment.

Neural sources underlying the magnetic fields and electric potentials were estimated using the L2-Minimum-Norm approach (L2-MNE; [49]) with a spherical shell consisting of 350 evenly distributed dipole pairs (MEG) or dipole triples (EEG) as the source model^1^. A source shell radius of 87% of the individually fitted head radius was chosen, which roughly corresponds to the grey matter depth. Leadfield matrices were calculated for all participants and conditions using a Tikhonov regularization parameter lambda of 0.1 for MEG and 0.2 for EEG data. The estimated neural source activity was calculated as the vector length of each dipole. Topographic maps displaying the direction-independent current dipole activity [50] were calculated for each participant, condition, and time point based on averaged magnetic/electric field distributions and individual sensor positions.

### Statistical analysis

Clinical data (Table 1) and behavioral tests (IQ, Touch Test, BSE) were compared between groups (AN vs. HC) using independent samples t-tests. Experimental behavioral data (deviant count, stimulus discrimination) was analyzed using mixed ANOVAs with the between-subject factor group (AN, HC) and the within-subject factor subtask (easy, difficult).

To analyze EMEG data, mixed ANOVAs were performed separately for the MEG-based and EEG-based estimated neural source activity, and separately for each neural source and each time point. These ANOVAs comprised the between-subject factor group (AN, HC) and the within-subject factors subtask (easy, difficult) and oddball (standard, deviant). Amplitudes of estimated neural source activity as well as corresponding effects are known to be considerably larger in late compared to early time intervals, and thus, large later effects can mask relevant effects in earlier time intervals [51]. To avoid this, time intervals of interest (TOIs) were defined from 40–60 ms, 80–250 ms, and 250–550 ms. Non-parametric cluster-based permutation tests [52] were performed to correct for multiple testing and to determine reliably significant spatiotemporal clusters (details in Supplement 2). For visualization, the revealed clusters were projected on a standard 3D brain model.

Additionally, possible associations between clinical, behavioural and neural data were addressed with exploratory correlation analyses using Pearson correlation coefficients (Supplement 5).

Pre-processing and analysis of EMEG data were done using the MATLAB-based software EMEGS version 3.1 (emegs.org; [53]). Statistical analysis was performed using IBM SPSS 29. All analyses adopted a significance level of α = 0.05 (except a first-level cluster criterion of α = 0.01, Supplement 2). Whenever applicable and necessary, F-statistics were Greenhouse Geisser corrected and t-statistics were Welch corrected.

## Results

### Sample characteristics

We recruited 40 AN patients and 59 HC participants in the context of a larger study including clinical data and two experimental paradigms during EMEG recording [45]. For the present analyses, we selected all participants who completed the tactile EMEG paradigm (AN = 36, HC = 41). To maximize statistical power, all available data was included. This led to slightly different sample sizes for the analyses of different data sources, i.e. most clinical and behavioral data was available in the full sample, but data quality control of EMEG data revealed smaller sample sizes (AN = 34, HC = 34; Supplement 3).

**Fig. 2:**
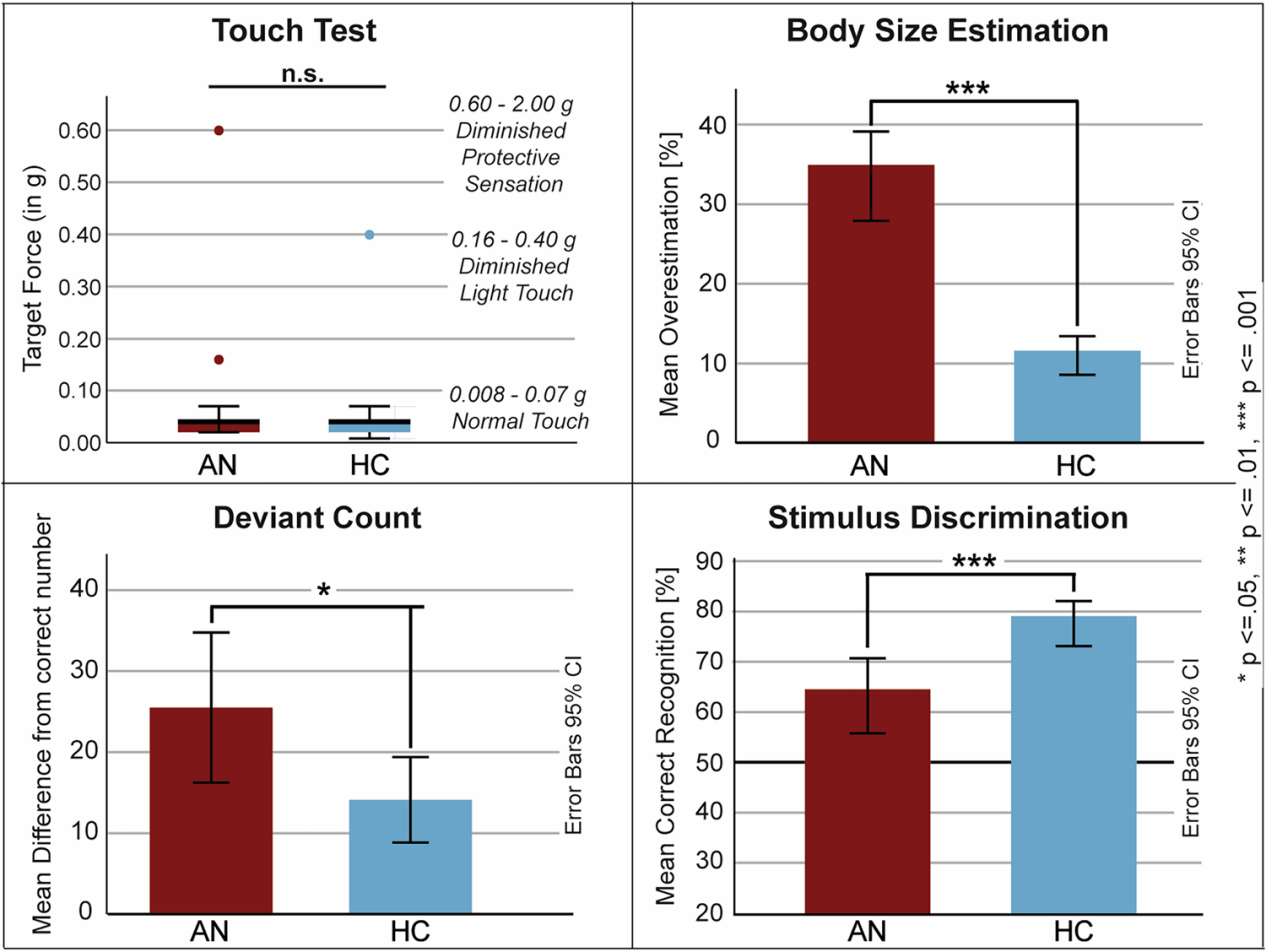
Results of the behavioral tasks. Missing data: Data of *n* = 3 AN patients is missing in the touch test and the deviant count task, data of *n* = 1 AN patient is missing in the body size estimation task and data of *n* = 2 AN patients and *n* = 1 HC participant is missing in the stimulus discrimination task.

The clinical characterization (Table 1) confirmed both the planned group matching regarding age, IQ, and handedness and the expected group differences regarding BMI and psychopathology, including both body dissatisfaction and body size overestimation (see Fig. 2). At the time of participation, patients had been in inpatient treatment for an average of 31.72 days. Further clinical details of the AN patient sample are presented in Table 1.

### Behavioral data

#### Body size estimation

AN patients overestimated their body size more than HC participants, as AN patients had larger mean BPIs (M = 135.36, SD = 17.13) than HC (M = 111.28, SD = 7.37), t(44,65) = 7.73, *p* < 0.001, d = 1.88.

#### Touch test

AN patients and HC participants had similar tactile perception thresholds *t*(72) = 0.785, *p* = 0.218, *d* = 0.18. In both groups, most participants had tactile perception thresholds within the normal range (0.07 g; AN group: 90,9%, HC group: 97.6%).

#### Deviant count

AN patients performed worse counting deviant tactile patterns than HC, indicated by larger differences of the counted vs. correct number of deviants, main effect of group: F(1, 72) = 5.38, *p* = 0.023, partial η2 = 0.070. Moreover, all participants performed better in the easy vs. difficult subtask, main effect of subtask: F(1, 72) = 15.57, *p* < 0.001, partial η2 = 0.178. The group × subtask interaction was not significant, F(1, 72) = 0.02, *p* = 0.897, partial η² = 0.000.

#### Tactile stimulus discrimination

AN patients also performed worse discriminating tactile patterns than HC, indicated by a lower proportion of correct answers, main effect of group: F(1, 72) = 12.00, *p* < 0.001, partial η2 = 0.143. Again, all participants performed better in the easy vs. difficult subtask, main effect of subtask: F(1, 72) = 14.71, *p* < 0.001, partial η2 = 0.170. The group × subtask interaction was not significant, F(1, 72) = 1.20, *p* = 0.277, partial η² = 0.016.

### EMEG data

#### Tactile oddball effect

**Fig. 3:**
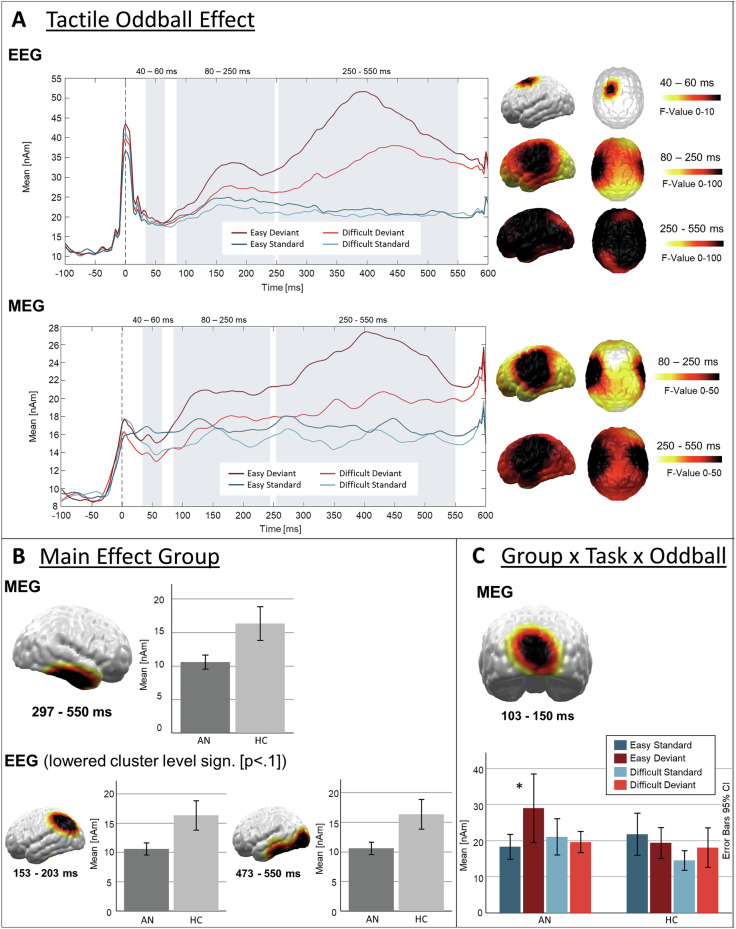
Neural responses evoked by the tactile Oddball paradigm. **A** Mean plot of the time course of estimated neural activity in response to each of the 4 tactile patterns (left) and spatio-temporal clusters with significant tactile oddball effects (right). Relatively increased processing of the deviant patterns was observed for both the easy and difficult subtask and was visible based on event related electric potentials (EEG) and event related magnetic fields (MEG). The distinct peak at the time of tactile stimulation (time = 0) reflects an electric artefact of the braille device which mainly affected the EEG and to lesser extent the MEG. The fact that the artefact develops even before stimulus onset is consequent upon the smoothing effect of the applied digital zero-phase forward-backward low-pass filter. **B** Relatively reduced neural activation in AN patients could be shown at inferior temporal regions based on MEG and EEG data, though at different hemispheres. EEG data additionally revealed a similar but earlier effect in the left PPC. Note that EEG based main effects of group were only visible with a lowered significance threshold of *p* < 0.1 regarding the first-level criterion (selection of data entering the cluster permutation tests) but met the second-level criterion of *p* < 0.05 (details on the non-parametric cluster-based permutation tests are reported in Supplement 2). **C** In a mid-latency occipital cluster MEG data revealed an oddball effect specifically in AN patients in the easy subtask.

The expected tactile oddball effect with stronger estimated neural source activity (in the following neural activity) in response to deviant vs. standard tactile patterns was observed for both the easy and difficult condition and across EEG and MEG data (Fig. 3A). A first EEG-based cluster showing a main effect of oddball occurred at 40–57 ms and was located at the contralateral somatosensory cortex, *p*-cluster = 0.003; F(1, 66) = 16.72, *p* < 0.001, partial η2 = 0.202. A second EEG-based cluster for the main effect of oddball was observed across the mid-latency TOI (80–250 ms) and covered the bilateral somatosensory cortex and adjacent regions in the frontal, parietal and temporal cortex, p-cluster < 0.001; F(1, 66) = 102.95, *p*  < 0.001, partial η2 = 0.609. Finally, an EEG-based cluster showing a main effect of oddball was found covering the late TOI (250–550 ms) and the whole brain, *p*-cluster < 0.001; F(1, 66) = 135.09, *p* < 0.001, partial η2 = 0.672.

There was no MEG-based cluster showing a main effect of oddball in the early TOI (40–60 ms). However, in the following time intervals MEG-based clusters closely mirrored the above EEG-based clusters. A MEG-based mid-latency (80–250 ms) cluster showing a main effect of oddball was located at the bilateral somatosensory cortex and adjacent regions in the frontal, parietal and temporal cortex, *p*-cluster < 0.001; F(1, 66) = 39.55, *p* < 0.001, partial η2 = 0.375, and a late (250–550 ms) MEG-based cluster covered the whole brain, *p*-cluster < 0.001; F(1, 66) = 72.15, *p* < 0.001, partial η2 = 0.522.

#### Main effect subtask and oddball x subtask interaction

EMEG data were modulated by task difficulty, indicated by clusters showing a main effect of subtask. Such clusters occurred in all TOIs and were widely distributed across the brain. In all of these clusters, neural activity was higher in the easy vs. difficult subtask. Consistently, the oddball effect was more pronounced in the easy vs. difficult subtask, as indicated by an oddball x subtask interaction in EEG- and MEG-based mid- and late-latency clusters primarily in the right and left dorsolateral prefrontal, and left parietal and temporal cortices. A detailed description and figures of these effects are presented in Supplement 4.

#### AN-specific effects

AN patients and HC participants did not reliably differ with respect to the overall oddball effect, as no spatio-temporal clusters were found showing a group x oddball interaction. However, in the mid-latency TOI (103 - 150 ms) a MEG-based cluster located in the occipital cortex indicated a group x oddball x subtask interaction, p-cluster = 0.031; F(1, 66) = 12.20, *p* < 0.001, partial η2 = 0.156 (Fig. 3C). In this cluster, an oddball effect with stronger neural responses to the deviant vs. standard tactile pattern was observed in the easy subtask and in AN patients only, post-hoc t-test deviant vs standard (easy, AN): t(33) = 2.47, *p* = 0.019, d = 0.42 (all other post hoc t-tests n.s.).

Moreover, AN patients showed overall lower neural activity than HC participants (Fig. 3B). More specifically, there was a MEG-based cluster showing a main effect of group in the late TOI (297 - 550 ms) located in the right inferior temporal cortex, *p*-cluster = 0.039; F(1, 66) = 18.74, *p* < 0.001, partial η2 = 0.221. Using a lowered cluster level significance threshold of *p* < 0.1, similar effects were found in the EEG data, though in the contralateral hemisphere. One EEG-based cluster showing the same main effect of group (AN < HC) occurred in the late TOI (473 – 550 ms) in the left inferior temporal cortex, *p*-cluster = 0.093; F(1, 66) = 15.27, *p* < 0.001, partial η2 = 0.188. A second EEG-based cluster showing a main effect of group was observed in the mid-latency TOI (153–203 ms) in the left posterior parietal cortex (PPC), *p*-cluster = 0.097; F(1, 66) = 14.90, *p* < 0.001, partial η2 = 0.184.

#### Exploratory correlation analyses

Exploratory correlation analyses yielded predominantly no to weak effects; however, the overall pattern largely pointed in the expected direction. The most robust findings were correlations between poorer tactile performance, particularly in the deviant count task, and both reduced oddball effect (deviant>standard) in distributed brain regions and more pronounced cognitive-affective BID (Supplement 5).

## Discussion

This study investigated behavioral and neurophysiological correlates of tactile perception in adolescents with AN. As expected, our findings demonstrate pronounced BID in AN, as evidenced by elevated BSQ and BSE scores, reflecting distortions in the cognitive-affective and perceptual dimensions of BID, respectively. AN patients and HC participants did not differ regarding their tactile perception thresholds. However, AN patients performed worse in two tasks requiring the differentiation of tactile patterns (deviant count and stimulus discrimination). At the same time, neural differentiation of tactile patterns appeared preserved in AN patients. Instead, we observed reduced neural activity in AN patients compared to HC in the right temporal cortex (MEG) and, at trend level, also in left temporal cortex and PPC (EEG).

The findings are consistent with previous studies that have found tactile deficits in AN [13, 28, 29], [33]. Importantly, in contrast to previous studies focusing on emotionally salient body parts [14], the present work tested tactile perception on the fingertip. Some authors assume that there are no differences in tactile tasks between AN patients and HC when cognitive-affective influences are minimized (e. g., [30]). However, others have reported alterations in tactile processing under emotionally neutral conditions [31, 54–56]. Thus, our results are in line with previous evidence that AN patients show deficits in neutral, relatively basic tactile perception tasks in which negative thoughts and feelings are not expected to greatly affect performance. Nevertheless, exploratory correlations showed a potential relationship of tactile task performance with cognitive-affective BID (Supplement 5), which should be explored in future studies.

Notably, neural differentiation of tactile stimuli was preserved in the AN sample, as indicated by early-onset, comparable oddball effects in both groups (Fig. 3A). Instead, the AN group showed reduced neural activity in mid- and late-latency TOIs, consistent with the expected reduced PPC activity during later stages of multisensory integration. However, this effect was not confined to the PPC and only reached trend level there, but was more pronounced in inferior temporal regions associated with higher-order visual (body) processing. The hemispheric distribution of these effects was unexpected and may reflect methodological factors and contralateral left-hemisphere involvement during right-sided stimulation (see Supplement 7).

Functional changes in inferior temporal regions have been reported previously in AN, though primarily as hyperactivations during visual body image processing tasks [24]. To contextualize the involvement of higher-order visual regions here, the three-dimensional structural model of body representations [57] provides a useful framework. Beyond multisensory body representations associated with the PPC, the model distinguishes between body representations derived from different sensory sources, each supporting specific functional demands. We propose that identifying tactile patterns beyond basic tactile discrimination requires access to different mental body representations, including visual representations in inferior temporal cortex. Participants may have relied on visual representations of their fingertip to map tactile input onto the body. This is consistent with the observation of the oddball effect not only in somatosensory but also in parietal and temporal regions (Fig. 3A). Reduced inferior temporal activity in AN may reflect diminished recruitment of such visual body representations. Exploratory correlations (warranting cautious interpretation; Supplement 5) further suggest an association between poorer deviant count performance and reduced oddball effect in the inferior temporal cortex (among other regions).

The assumption of multiple body representations, which may be specific to particular sensory modalities [57], is also useful for interpreting the AN- and subtask-specific oddball effect observed in the occipital cortex (Fig. 3C). Due to the limited availability of more abstract visuospatial body representations, AN patients may have relied on object-like visual imagery represented in primary visual regions [58]. Such a pattern would be consistent with the notion of inefficient multisensory integration in AN, whereby reduced recruitment of higher-order association areas (i.e., temporal and parietal regions) is accompanied by a relative overreliance on unimodal sensory (i.e., occipital) regions. However, this interpretation remains speculative.

Taken together, these findings are consistent with evidence for impaired multisensory integration in AN [21, 26, 59]. In the context of our tactile paradigm, this likely involves the integration of tactile input with both basic tactile and visual, as well as higher-order multisensory body representations. Given preserved neural differentiation alongside poorer behavioral performance, AN patients may exhibit deficits in integrating correctly encoded sensory information into a coherent higher-order body representation, thereby impairing behavioral performance.

## Limitations

A first limitation is the likely presence of brain atrophy in underweight AN patients [60] which might have distorted the amplitudes of EEG-based scalp ERPs [61], as these are strongly influenced by the proportion of different tissues and liquids in the brain [62]. Thus, the observed hypoactivity (Fig. 3B) might, at least in part, reflect AN-related brain atrophy. However, this effect was even more robust in the MEG data, which is almost unaffected by volume conduction effects, speaking against a pure methodological artifact. Moreover, we did not observe neural hypoactivity in a largely overlapping AN sample during a visual task [45], suggesting some specificity to the tactile task. Following this line of thought, AN patients performed as well as HC in both a visual behavioral task ([45], Supplement 6) and an intelligence test. This speaks against a general cognitive performance deficit and suggests a relative specificity of the observed tactile deficits. Another limitation concerns the limited sample size. To keep the sample as large as possible, we decided to include all valid data. One disadvantage of this decision is that not all data sets are based on the same participants, reducing the comparability of the different data sources.

## Conclusions

The findings suggest that, although tactile stimulus discrimination is unaffected on a neural level, AN patients have difficulties discriminating tactile stimuli behaviorally. This performance deficit is accompanied by reduced neural activity in temporal and by trend also PPC regions in mid-latency and late time intervals. We suggest that this combination of effects reflects aberrant multisensory integration - a process that is crucial for the coherence of body representation. This neuronal dysfunction could contribute to maintaining or reinforcing the perceptual component of BID.

This work underlines the potential of tactile perception as a candidate for a mechanism-based optimization of the BID treatment in AN. One goal might be to improve tactile body awareness and the integration of tactile perceptions into a coherent multisensory body representation. A promising approach in this direction, for example, is an adolescent AN-specific body psychotherapy concept based on Concentrative Movement Therapy [11]. This approach comprises practical training in conscious tactile, interoceptive, and proprioceptive body perception in a first step, which is linked to the cognitive-affective component of body image, including self-opening in interpersonal relationships, in a second step.

## Supplementary information

Supplemental Material

## Data Availability

The datasets generated for this study are available on request to the corresponding author.

## Footnotes

1 Note that source estimations of EEG data were based on 57 or 80 EEG channels, respectively, but both resulted in 350 dipole triplets. Because in a spherical model radial sources do not generate measurable magnetic fields outside the head, dipole pairs (azimuthal and polar directions only) were estimated for MEG, and dipole triplets (azimuthal, polar, and radial directions) were estimated for EEG.
