# Supplementary material for: Tactile stimulus discrimination in adolescents with anorexia nervosa: a behavioral and neurophysiological study
Source: Transl Psychiatry. 2026 Jul 23;16:379. doi: 10.1038/s41398-026-04264-3 (PMC13400626; doi:10.1038/s41398-026-04264-3)
Supplement: Supplementary file 1 — Supplemental Material [file 41398_2026_4264_MOESM1_ESM.docx]

# Suppements

**Supplement 1: Touch Test**

The Touch Test uses so-called Semmes-Weinstein monofilaments made of nylon. The monofilaments vary in thickness and are labelled with different numbers between 0.008 and 0.6. These numbers indicate how much force (in grams) is required to bend each filament. Participants were asked to sit quietly and place their right hand on a table with the inside facing up. The participants' eyes were covered with a blindfold. The monofilaments were pressed one at a time against the skin at the participant's fingertip for approximately 1.5 s. Subjects were asked to say "yes" when they felt a stimulus. Monofilaments were presented randomly. If one monofilament was reliably detected and the monofilament with the next lower gram value was not, the test was completed. The gram of the thinnest monofilament that was reliably detected was noted and this information served as the value for the tactile perception threshold.

**Supplement 2: Details on the non-parametric cluster-based permutation tests**

Non-parametric cluster-based permutation tests based on the work by (Maris & Oostenveld, 2007) were performed to correct for multiple testing. Reliably significant spatiotemporal clusters were determined as follows: First, within each specified time interval, estimated source activities were considered for further analysis only if they had significant ANOVA effects with a p-value of <.01 (first-level criterion). Temporally and spatially adjacent significant F-values of the underlying sources were summed to form cluster masses. Cluster masses were evaluated against a distribution of 1000 random permutations of the same datasets (for each permutation, the biggest identified first-level significant cluster mass was considered). Clusters were only considered significant if their cluster mass surpassed the 950th highest cluster mass of the random distribution, equivalent to p <.05 (second-level criterion). The estimated source activity data within each identified spatiotemporal cluster were extracted and further analysed. The results of the mixed ANOVAs were supplemented by post hoc t-tests.


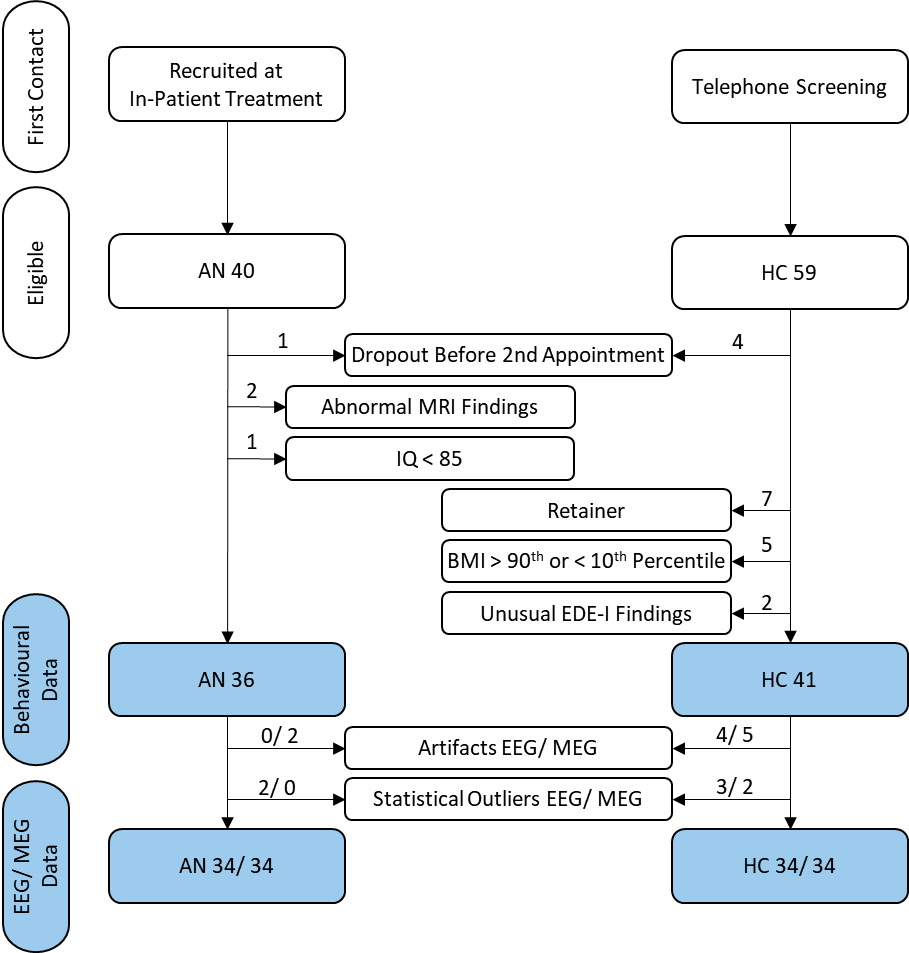
**Supplement 3: Recruitment and Sample Sizes**

Flow Chart visualizing the recruitment process. Note that EEG and MEG analyses each comprise 34 AN patients and 34 HC participants. However, of these N = 32 AN patients and N = 29 HC participants are the same, while for N = 2 AN patients and N = 5 HC participants only EEG or MEG data were available.

**Supplement 4: EMEG Main Effect Subtask and Oddball x Subtask Interaction**

**Main Effekt Subtask**

A first EEG-based cluster showing a main effect of Task was found mid-latency (80-250 ms) and located mostly in the left, contralateral somatosensory cortex and adjacent regions, p of cluster < .001; F(1, 66) = 51.43, p < .001, partial η2 = .438, and a second EEG-based cluster was observed in the late time interval (250-550 ms), and covered almost the whole brain, except orbitofrontal and posterior temporal regions, p of cluster < .001; F(1, 66) = 61.91, p < .001, partial η2 = .484. Three MEG-based clusters showing the above main effect of task occurred already during the early (40-60 ms) time interval, and were located in the right temporoparietal cortex, p of cluster .001, F(1, 66) = 11.60, p = .001, partial η2 = .149, the right inferior frontal region, p of cluster .011, F(1, 66) = 16.09, p < .001, partial η2 = .196, and in the left temporoparietal cortex, p of cluster .025, F(1, 66) = 9.24, p = .003, partial η2 = .123. A mid-latency (80-250 ms) MEG-based cluster was located in the left temporoparietal cortex and the the right inferior frontal region, p of cluster < .001; F (1, 66) = 36.52, p < .001, partial η2 = .356. Finally, a late (250-550 ms) cluster was located in the same regions but extended also to bilateral posterior temporal regions, 250 – 527 ms: p of clusters < .001; F (1, 66) = 28.79, p < .001, partial η2 = .304.

**Oddball x Subtask Interaction**

In the EEG data, a mid-latency interaction cluster was located in dorsal parietal and frontal regions, 80 - 213 ms: p of cluster < .001; F(1, 66) = 32.03, p < .001, partial η2 = .327. In the left hemisphere, the cluster was extended to the dorsal part of the temporal lobe, and the strongest effects were found in the ventral part of the somatosensory cortex and surrounding areas. In the late time interval, the cluster covered almost the entire brain, except smaller regions in the temporal lobe, 250 - 533 ms: p of cluster < .001; F(1, 66) = 51.75, p < .001, partial η2 = .440. The strongest effect was found on the right, at the transition between frontal and parietal lobe and in the anterior part of the right temporal lobe. Post-hoc two-sided t-tests revealed that neuronal activity in response to the *standard* did not differ significantly between the easy and difficult subtasks, 80 - 213 ms: t(67) = 1.68, p = .097, d = 0.20; 250 - 533 ms: t(67) = 1.22, p = .228, d = 0.15. However, the neuronal activity in response to the oddball was stronger in the easy subtask than in the difficult subtask, 80 - 213 ms: t(67) = 7. 04, p < .001, d = 0.85; 250 - 533 ms: t(67) = 7.87, p < .001, d = 0.95. The oddball effect - i.e. the stronger neuronal response to the *oddball* compared to the *standard* - was therefore more pronounced in the easy subtask in both time intervals. However, a significant *oddball* effect occurred in both time intervals in both subtasks, 80 - 213 ms: Easy: t(67) = 10.00, p < .001, d = 1.21; Difficult: t(67) = 6.10, p < .001, d = 0.74; 250 - 550 ms: Easy: t(67) = 12.26, p < .001, d = 1.49; Difficult: t(67) = 7.22, p < .001, d = 0.88.

In the MEG data, there were two mid-latency clusters. One cluster localized in the left lateral temporal and inferior parietal cortex, 80 – 180 ms: p of cluster < .001; F(1, 66) = 37.27, p < .001, partial η2 = .361. The other cluster localized in the right inferior parietal cortex, 130 - 197 ms: p of cluster .017; F(1, 66) = 22.75, p < .001, partial η2 = .256. In the late time interval, an interaction cluster was localized mainly in bilateral frontal and temporal cortices, 320 – 543 ms: p of cluster < .001; F(1, 66) = 24.79, p < .001, partial η2 = .273.

Post-hoc two-sided t-tests revealed that the neuronal activity in response to the *standard* did not differ significantly between the easy and difficult subtasks, 80 – 180 ms: t(67) = 0.66, p = .512, d = 0.08; 130 – 197 ms: t(67) = 0.66, p = .513, d = 0.08; 320 – 543 ms: t(67) = 0.29, p = .774, d = 0.04. However, the neuronal activity in response to the oddball was stronger in the easy subtask than in the difficult subtask, 80 – 180 ms: t(67) = 7.49, p <.001, d = 0.90; 130 – 197 ms: t(67) = 4.67, p < .001, d = 0.57; 320 – 543 ms: t(67) = 5.34, p < .001, d = 0.65. The *oddball* effect was therefore more pronounced in the easy subtask in both time ranges (and both clusters of the second time range).

However, a significant *oddball* effect occurred in two of the three clusters, 80 – 180 ms: Easy: t(67) = 9.80, p < .001, d = 1.19; Difficult: t(67) = 2.75, p = .008, d = 0.33; 320 – 543 ms: Easy: t(67) = 8.22, p < .001, d = 1.00; Difficult: t(67) = 3.39, p < .001, d = 0.41. In the second cluster of the second time range (130 – 197 ms) there was only an oddball effect in the easy subtask, t(67) = 6.05, p < .001, d = 0.74, but not in the difficult subtask, t(67) = 1.28, p = .204, d = 0.16.

**Supplementary Figure 4**

**
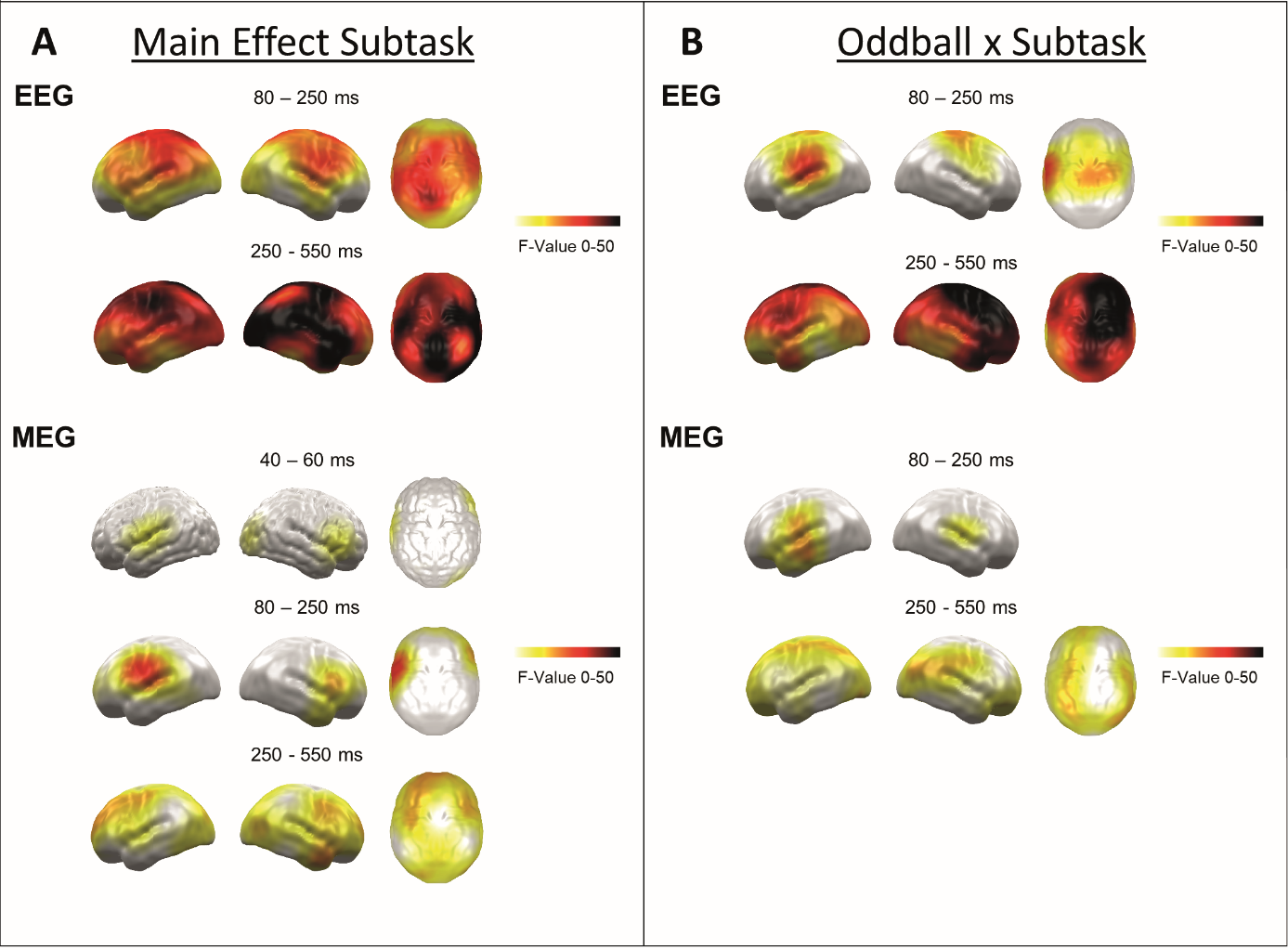
**

**Supplement 5: Exploratory Correlation Analysis**

**Exploratory correlations of tactile task performance with measures of BID**

Poorer tactile performance (deviant count: higher values = poorer performance; tactile stimulus discrimination: lower values = poorer performance) was correlated with higher levels of body image disturbance (BID) across groups. However, this effect was not consistently replicated in separate analyses within the AN and HC groups. Only the association between deviant count and EDI-C BD reached trend level in both groups. Accordingly, we interpret these findings with appropriate caution.

**Tactile performance all participants:**

|  | | BSQ | EDI-C DT | EDI-C BD | BSE |
| --- | --- | --- | --- | --- | --- |
| Deviant Count | Pearson Correlation | **.349** | **.338** | **.396** | .070 |
|  | Sig. (2-sided) | **.002** | **.003** | **.000** | .554 |
|  | N | 73 | 74 | 74 | 73 |
| Tactile Stimulus Discrimination | Pearson Correlation | **-.387** | **-.440** | **-.387** | **-.319** |
|  | Sig. (2-sided) | **.001** | **.000** | **.001** | **.006** |
|  | N | 73 | 74 | 74 | 73 |

**Tactile performance separate analyses of AN and HC groups:**

| **AN Group** | | BSQ | EDI-C DT | EDI-C BD | BSE |
| --- | --- | --- | --- | --- | --- |
| Deviant Count | Pearson Correlation | .283 | .143 | **.330** | -.223 |
|  | Sig. (2-sided) | .110 | .429 | **.061** | .220 |
|  | N | 33 | 33 | 33 | 32 |
| Tactile Stimulus Discrimination | Pearson Correlation | -.261 | -.259 | -.266 | -.160 |
|  | Sig. (2-sided) | .137 | .138 | .129 | .375 |
|  | N | 34 | 34 | 34 | 33 |

| **HC Group** | | BSQ | EDI-C DT | EDI-C BD | BSE |
| --- | --- | --- | --- | --- | --- |
| Deviant Count | Pearson Correlation | .172 | **.501** | **.294** | .143 |
|  | Sig. (2-sided) | .290 | **.001** | **.062** | .374 |
|  | N | 40 | 41 | 41 | 41 |
| Tactile Stimulus Discrimination | Pearson Correlation | .070 | -.233 | -.049 | .073 |
|  | Sig. (2-sided) | .670 | .148 | .765 | .656 |
|  | N | 39 | 40 | 40 | 40 |

**Exploratory correlations of the neural oddball effect (deviant minus standard) in clusters showing a respective main effect with measures of BID**

Analyses across participants revealed no significant effects, and separate analyses within AN and HC groups revealed a small number of trends, but no robust effects. If anything, a smaller neural oddball effect tended to be associated with more pronounced BID.

**MEG all participants:**

|  | | BSQ | EDI-C DT | EDI-C BD | BSE |
| --- | --- | --- | --- | --- | --- |
| MEG 80-250 Deviant>Standard | Pearson Correlation | .070 | .139 | -.021 | .128 |
|  | Sig. (2-sided) | .571 | .257 | .864 | .301 |
|  | N | 67 | 68 | 68 | 67 |
| MEG 250-550 Deviant>Standard | Pearson Correlation | -.076 | -.104 | -.146 | -.092 |
|  | Sig. (2-sided) | .541 | .397 | .234 | .458 |
|  | N | 67 | 68 | 68 | 67 |

**MEG separate analyses of AN and HC groups:**

| **AN Group** | | BSQ | EDI-C DT | EDI-C BD | BSE |
| --- | --- | --- | --- | --- | --- |
| MEG 80-250 Deviant>Standard | Pearson Correlation | -.003 | .069 | -.144 | .092 |
|  | Sig. (2-sided) | .987 | .698 | .417 | .611 |
|  | N | 34 | 34 | 34 | 33 |
| MEG 250-550 Deviant>Standard | Pearson Correlation | -.265 | -.268 | **-.308** | -.079 |
|  | Sig. (2-sided) | .130 | .126 | **.077** | .663 |
|  | N | 34 | 34 | 34 | 33 |

| **HC Group** | | BSQ | EDI-C DT | EDI-C BD | BSE |
| --- | --- | --- | --- | --- | --- |
| MEG 80-250 Deviant>Standard | Pearson Correlation | -.214 | -.123 | **-.287** | -.175 |
|  | Sig. (2-sided) | .233 | .490 | **.100** | .322 |
|  | N | 33 | 34 | 34 | 34 |
| MEG 250-550 Deviant>Standard | Pearson Correlation | -.053 | -.167 | -.182 | **-.407** |
|  | Sig. (2-sided) | .770 | .345 | .304 | **.017** |
|  | N | 33 | 34 | 34 | 34 |

**EEG all participants:**

|  | | BSQ | EDI-C DT | EDI-C BD | BSE |
| --- | --- | --- | --- | --- | --- |
| EEG 40-57 Deviant>Standard | Pearson Correlation | .108 | .116 | .080 | .196 |
|  | Sig. (2-sided) | .383 | .345 | .515 | .111 |
|  | N | 67 | 68 | 68 | 67 |
| EEG 80-250 Deviant>Standard | Pearson Correlation | -.009 | -.036 | -.098 | .037 |
|  | Sig. (2-sided) | .941 | .773 | .426 | .766 |
|  | N | 67 | 68 | 68 | 67 |
| EEG 250-550 Deviant>Standard | Pearson Correlation | -.104 | -.106 | **-.203** | -.037 |
|  | Sig. (2-sided) | .400 | .388 | **.098** | .769 |
|  | N | 67 | 68 | 68 | 67 |

**EEG separate analyses of AN and HC groups:**

| **AN Group** | | BSQ | EDI-C DT | EDI-C BD | BSE |
| --- | --- | --- | --- | --- | --- |
| EEG 40-57 Deviant>Standard | Pearson Correlation | .084 | .122 | .109 | .244 |
|  | Sig. (2-sided) | .637 | .490 | .539 | .170 |
|  | N | 34 | 34 | 34 | 33 |
| EEG 80-250 Deviant>Standard | Pearson Correlation | .043 | .090 | -.070 | .121 |
|  | Sig. (2-sided) | .808 | .614 | .696 | .502 |
|  | N | 34 | 34 | 34 | 33 |
| EEG 250-550 Deviant>Standard | Pearson Correlation | -.076 | .055 | -.123 | .127 |
|  | Sig. (2-sided) | .670 | .756 | .489 | .482 |
|  | N | 34 | 34 | 34 | 33 |

| **HC Group** | | BSQ | EDI-C DT | EDI-C BD | BSE |
| --- | --- | --- | --- | --- | --- |
| EEG 40-57 Deviant>Standard | Pearson Correlation | -.161 | -.246 | -.188 | -.036 |
|  | Sig. (2-sided) | .370 | .161 | .287 | .841 |
|  | N | 33 | 34 | 34 | 34 |
| EEG 80-250 Deviant>Standard | Pearson Correlation | .018 | -.116 | -.084 | .106 |
|  | Sig. (2-sided) | .921 | .514 | .637 | .553 |
|  | N | 33 | 34 | 34 | 34 |
| EEG 250-550 Deviant>Standard | Pearson Correlation | -.218 | **-.393** | **-.328** | -.226 |
|  | Sig. (2-sided) | .223 | **.022** | **.059** | .198 |
|  | N | 33 | 34 | 34 | 34 |

**Exploratory correlations of neural activity and neural oddball effect (deviant minus standard) in clusters showing a main effect of group with measures of BID**

Analyses across participants revealed negative correlations between overall neural activity and in part also the neural oddball effect (deviant minus standard) in clusters showing a main effect of group. Thus, lower neural activity and in part lower difference activity (reduced oddball effect) was associated with more pronounced BID. However, this was largely not replicated in separate analyses within the AN and HC groups. This suggests that the correlations may be spurious and largely driven by the relevant between-group differences in the respective variables.

**MEG all participants:**

|  | | BSQ | EDI-C DT | EDI-C BD | BSE |
| --- | --- | --- | --- | --- | --- |
| MEG 297-550 Mean | Pearson Correlation | **-.386** | **-.431** | **-.400** | **-.297** |
|  | Sig. (2-sided) | **.001** | **<.001** | **<.001** | **.015** |
|  | N | 67 | 68 | 68 | 67 |
| MEG 297-550 Deviant>Standard | Pearson Correlation | -.092 | -.088 | -.135 | -.101 |
|  | Sig. (2-sided) | .457 | .478 | .271 | .418 |
|  | N | 67 | 68 | 68 | 67 |

**MEG separate analyses of AN and HC groups:**

| **AN Group** | | BSQ | EDI-C DT | EDI-C BD | BSE |
| --- | --- | --- | --- | --- | --- |
| MEG 297-550 Mean | Pearson Correlation | -.202 | -.234 | -.205 | -.024 |
|  | Sig. (2-sided) | .252 | .182 | .244 | .896 |
|  | N | 34 | 34 | 34 | 33 |
| MEG 297-550 Deviant>Standard | Pearson Correlation | -.217 | -.198 | **-.301** | -.062 |
|  | Sig. (2-sided) | .217 | .261 | **.083** | .732 |
|  | N | 34 | 34 | 34 | 33 |

| **HC Group** | | BSQ | EDI-C DT | EDI-C BD | BSE |
| --- | --- | --- | --- | --- | --- |
| MEG 297-550 Mean | Pearson Correlation | -.038 | -.071 | -.069 | .077 |
|  | Sig. (2-sided) | .833 | .692 | .700 | .667 |
|  | N | 33 | 34 | 34 | 34 |
| MEG 297-550 Deviant>Standard | Pearson Correlation | -.137 | -.172 | -.165 | **-.300** |
|  | Sig. (2-sided) | .446 | .332 | .350 | **.084** |
|  | N | 33 | 34 | 34 | 34 |

**EEG all participants:**

|  | | BSQ | EDI-C DT | EDI-C BD | BSE |
| --- | --- | --- | --- | --- | --- |
| EEG 153-203 Mean | Pearson-Correlation | **-.399** | **-.412** | **-.414** | **-.348** |
|  | Sig. (2-sided) | **.001** | **.000** | **.000** | **.004** |
|  | N | 67 | 68 | 68 | 67 |
| EEG 153-203  Deviant >Standard | Pearson-Correlation | **-.205** | **-.220** | **-.238** | -.041 |
|  | Sig. (2-sided) | **.096** | **.072** | **.050** | .739 |
|  | N | 67 | 68 | 68 | 67 |
| EEG 473-550 Mean | Pearson-Correlation | **-.366** | **-.385** | **-.435** | **-.333** |
|  | Sig. (2-sided) | **.002** | **.001** | **.000** | **.006** |
|  | N | 67 | 68 | 68 | 67 |
| EEG 473-550  Deviant>Standard | Pearson-Correlation | **-.239** | **-.225** | **-.352** | -.174 |
|  | Sig. (2-sided) | **.052** | **.066** | **.003** | .160 |
|  | N | 67 | 68 | 68 | 67 |

**EEG separate analyses of AN and HC groups:**

| Group | | | BSQ | EDI-C DT | | | EDI-C BD | | BSE |
| --- | --- | --- | --- | --- | --- | --- | --- | --- | --- |
| AN | EEG 153-203  Mean | Pearson-Correlation | -.170 | -.154 | | | -.141 | | -.141 |
|  |  | Sig. (2-sided) | .335 | .384 | | | .428 | | .434 |
|  |  | N | 34 | 34 | | | 34 | | 33 |
|  | EEG 153-203  Deviant>Standard | Pearson-Correlation | -.079 | -.040 | | | -.135 | | .092 |
|  |  | Sig. (2-sided) | .655 | .823 | | | .448 | | .610 |
|  |  | N | 34 | 34 | | | 34 | | 33 |
|  | EEG 473-550  Mean | Pearson-Correlation | .081 | .119 | | | .020 | | .024 |
|  |  | Sig. (2-sided) | .650 | | .502 | .910 | | .894 | |
|  |  | N | 34 | | 34 | 34 | | 33 | |
|  | EEG 473-550  Deviant>Standard | Pearson-Correlation | -.102 | | .067 | -.117 | | .058 | |
|  |  | Sig. (2-sided) | .567 | | .708 | .510 | | .748 | |
|  |  | N | 34 | | 34 | 34 | | 33 | |
| HC | EEG 153-203 Mean | Pearson-Correlation | -.192 | | -.146 | -.189 | | -.078 | |
|  |  | Sig. (2-sided) | .285 | | .411 | .285 | | .660 | |
|  |  | N | 33 | | 34 | 34 | | 34 | |
|  | EEG 153-203  Deviant>Standard | Pearson-Correlation | -.180 | | **-.296** | -.162 | | .204 | |
|  |  | Sig. (2-sided) | .316 | | **.089** | .360 | | .246 | |
|  |  | N | 33 | | 34 | 34 | | 34 | |
|  | EEG 473-550 Mean | Pearson-Correlation | **-.368** | | **-.346** | **-.351** | | -.217 | |
|  |  | Sig. (2-sided) | **.035** | | **.045** | **.042** | | .218 | |
|  |  | N | 33 | | 34 | 34 | | 34 | |
|  | EEG 473-550  Deviant>Standard | Pearson-Correlation | -.281 | | **-.370** | **-.450** | | -.205 | |
|  |  | Sig. (2-sided) | .113 | | **.031** | **.008** | | .245 | |
|  |  | N | 33 | | 34 | 34 | | 34 | |

**Exploratory correlations of differential neural activity (deviant minus standard) in clusters showing an oddball effect with tactile task performance**

The most robust findings of the exploratory correlation analyses were observed for the association between the neural oddball effect (deviant minus standard) in respective clusters and performance in the tactile tasks. Across groups, significant correlations associating reduced neural differentiation with poorer deviant count performance were found in the late clusters (250-550 ms) in both MEG and EEG and in additionally in the mid-latency cluster (80-250 ms) in the EEG. Moreover, in the late EEG cluster, reduced neural differentiation was also associated with poorer tactile stimulus discrimination performance. All of these correlations proved relatively robust in that the effects remained significant in separate within-group analyses or were at least observable at a trend level. In light of the large number of uncorrected exploratory correlation analyses, we view these findings with appropriate caution. However, they may provide an indication that performance in the tactile discrimination tasks is mirrored in the observed neural activity.

**MEG all participants:**

|  | | Deviant Count | Tactile Stimulus Discrimination |
| --- | --- | --- | --- |
| MEG 80-250 Deviant>Standard | Pearson-Correlation | **-.236** | .030 |
|  | Sig. (2-sided) | **.058** | .812 |
|  | N | 65 | 65 |
| MEG 250-550 Deviant>Standard | Pearson-Correlation | **-.428** | .115 |
|  | Sig. (2-sided) | **<.001** | .360 |
|  | N | 65 | 65 |

**MEG separate analyses of AN and HC groups:**

| Group | | | Deviant Count | Tactile Stimulus Discrimination |
| --- | --- | --- | --- | --- |
| AN | MEG 80-250  Deviant>Standard | Pearson-Correlation | **-.312** | .086 |
|  |  | Sig. (2-sided) | **.087** | .641 |
|  |  | N | 31 | 32 |
|  | MEG 250-550  Deviant>Standard | Pearson-Correlation | **-.574** | .221 |
|  |  | Sig. (2-sided) | **.001** | .223 |
|  |  | N | 31 | 32 |
| HC | MEG 80-250  Deviant>Standard | Pearson-Correlation | -.243 | .148 |
|  |  | Sig. (2-sided) | .166 | .411 |
|  |  | N | 34 | 33 |
|  | MEG 250-550  Deviant>Standard | Pearson-Correlation | **-.308** | .035 |
|  |  | Sig. (2-sided) | **.076** | .846 |
|  |  | N | 34 | 33 |

**EEG all participants:**

|  | | Deviant Count | Tactile Stimulus Discrimination |
| --- | --- | --- | --- |
| EEG 40-57 Deviant>Standard | Pearson-Correlation | -.099 | -.110 |
|  | Sig. (2-sided) | .431 | .384 |
|  | N | 65 | 65 |
| EEG 80-250 Deviant>Standard | Pearson-Correlation | **-.364** | .160 |
|  | Sig. (2-sided) | **.003** | .203 |
|  | N | 65 | 65 |
| EEG 250-550 Deviant>Standard | Pearson-Correlation | **-.662** | **.358** |
|  | Sig. (2-sided) | **.000** | **.003** |
|  | N | 65 | 65 |

**EEG separate analyses of AN and HC groups:**

| Group | | | Deviant Count | Tactile Stimulus Discrimination |
| --- | --- | --- | --- | --- |
| AN | EEG 40-57  Deviant>Standard | Pearson-Correlation | -.025 | -.065 |
|  |  | Sig. (2-sided) | .892 | .724 |
|  |  | N | 31 | 32 |
|  | EEG 80-250  Deviant>Standard | Pearson-Correlation | **-.407** | .133 |
|  |  | Sig. (2-sided) | **.023** | .466 |
|  |  | N | 31 | 32 |
|  | EEG 250-550 Deviant>Standard | Pearson-Correlation | **-.726** | **.403** |
|  |  | Sig. (2-sided) | **.000** | **.022** |
|  |  | N | 31 | 32 |
| HC | EEG 40-57 Deviant>Standard | Pearson-Correlation | **-.330** | -.049 |
|  |  | Sig. (2-sided) | **.057** | .784 |
|  |  | N | 34 | 33 |
|  | EEG 80-250 Deviant>Standard | Pearson-Correlation | **-.307** | .196 |
|  |  | Sig. (2-sided) | **.077** | .273 |
|  |  | N | 34 | 33 |
|  | EEG 250-550 Deviant>Standard | Pearson-Correlation | **-.619** | **.304** |
|  |  | Sig. (2-sided) | **.000** | **.086** |
|  |  | N | 34 | 33 |

**Exploratory correlations of neural activity and neural oddball effect (deviant minus standard) in clusters showing a main effect of group with tactile task performance**

Across both groups, neural activity and the magnitude of the oddball effect in regions with reduced activation in the AN relative to the HC group were significantly associated with tactile task performance in several clusters. In all cases, reduced neural activity or reduced neural differentiation between deviant and standard stimuli was linked to poorer performance. In many cases, these effects were not robust and were no longer significant in separate within-group analyses. Notably, in one case, the correlation persisted: In a late EEG cluster (473-550 ms) showing a main effect of group, a smaller neural oddball effect (smaller difference between deviant and standard) was associated with poorer performance in the deviant count task, both across and within groups. In light of the many uncorrected exploratory correlation analyses conducted, we interpret this finding with the utmost caution. Still, it fits the general pattern that, where present, correlations were in the expected direction.

**MEG all participants:**

|  | | Deviant Count | Tactile Stimulus Discrimination |
| --- | --- | --- | --- |
| MEG Mean | Pearson-Correlation | **-.242** | **.297** |
|  | Sig. (2-sided) | **.052** | **.016** |
|  | N | 65 | 65 |
| MEG Deviant>Standard | Pearson-Correlation | **-.282** | .105 |
|  | Sig. (2-sided) | **.023** | .405 |
|  | N | 65 | 65 |

**MEG separate analyses of AN and HC groups:**

| Group | | | Deviant Count | Tactile Stimulus Discrimination |
| --- | --- | --- | --- | --- |
| AN | MEG Mean | Pearson-Correlation | **-.302** | .195 |
|  |  | Sig. (2-sided) | **.099** | .284 |
|  |  | N | 31 | 32 |
|  | MEG  Deviant>Standard | Pearson-Correlation | **-.545** | .197 |
|  |  | Sig. (2-sided) | **.002** | .280 |
|  |  | N | 31 | 32 |
| HC | MEG Mean | Pearson-Correlation | -.098 | .133 |
|  |  | Sig. (2-sided) | .583 | .459 |
|  |  | N | 34 | 33 |
|  | MEG  Deviant>Standard | Pearson-Correlation | -.236 | .086 |
|  |  | Sig. (2-sided) | .178 | .633 |
|  |  | N | 34 | 33 |

**EEG all participants:**

|  | | Deviant Count | Tactile Stimulus Discrimination |
| --- | --- | --- | --- |
| EEG 153-203  Mean | Pearson-Correlation | -.134 | .103 |
|  | Sig. (2-sided) | .287 | .414 |
|  | N | 65 | 65 |
| EEG 153-203  Deviant>Standard | Pearson-Correlation | **-.314** | **.274** |
|  | Sig. (2-sided) | **.011** | **.027** |
|  | N | 65 | 65 |
| EEG 473-550  Mean | Pearson-Correlation | **-.330** | **.335** |
|  | Sig. (2-sided) | **.007** | **.006** |
|  | N | 65 | 65 |
| EEG 473-550  Deviant>Standard | Pearson-Correlation | **-.543** | **.374** |
|  | Sig. (2-sided) | **.000** | **.002** |
|  | N | 65 | 65 |

**EEG separate analyses of AN and HC groups:**

| Group | | | Deviant Count | Tactile Stimulus Discrimination |
| --- | --- | --- | --- | --- |
| AN | EEG 153-203 Mean | Pearson-Correlation | -.095 | .033 |
|  |  | Sig. (2-sided) | .611 | .857 |
|  |  | N | 31 | 32 |
|  | EEG 153-203 Deviant>Standard | Pearson-Correlation | **-.363** | .245 |
|  |  | Sig. (2-sided) | **.045** | .177 |
|  |  | N | 31 | 32 |
|  | EEG 473-550 Mean | Pearson-Correlation | -.271 | .203 |
|  |  | Sig. (2-sided) | .140 | .266 |
|  |  | N | 31 | 32 |
|  | EEG 473-550 Deviant>Standard | Pearson-Correlation | **-.595** | **.351** |
|  |  | Sig. (2-sided) | **.000** | **.049** |
|  |  | N | 31 | 32 |
| HC | EEG 153-203 Mean | Pearson-Correlation | .053 | -.226 |
|  |  | Sig. (2-sided) | .766 | .207 |
|  |  | N | 34 | 33 |
|  | EEG 153-203 Deviant>Standard | Pearson-Correlation | -.190 | .243 |
|  |  | Sig. (2-sided) | .282 | .173 |
|  |  | N | 34 | 33 |
|  | EEG 473-550 Mean | Pearson-Correlation | -.267 | .211 |
|  |  | Sig. (2-sided) | .127 | .240 |
|  |  | N | 34 | 33 |
|  | EEG 473-550 Deviant>Standard | Pearson-Correlation | **-.519** | **.344** |
|  |  | Sig. (2-sided) | **.002** | **.050** |
|  |  | N | 34 | 33 |

**Supplement 6: Depictive Body Size Estimation Task**

As part of an overarching study protocol, participants in our study on tactile stimulus discrimination also completed a depictive body size estimation (BSE) paradigm. With the exception of one healthy participant, behavioural data on depictive body size estimation are available for all participants whose behavioural data on tactile perception (Deviant Count, Tactile Stimulus Discrimination) were analysed (AN = 36, HC = 40).


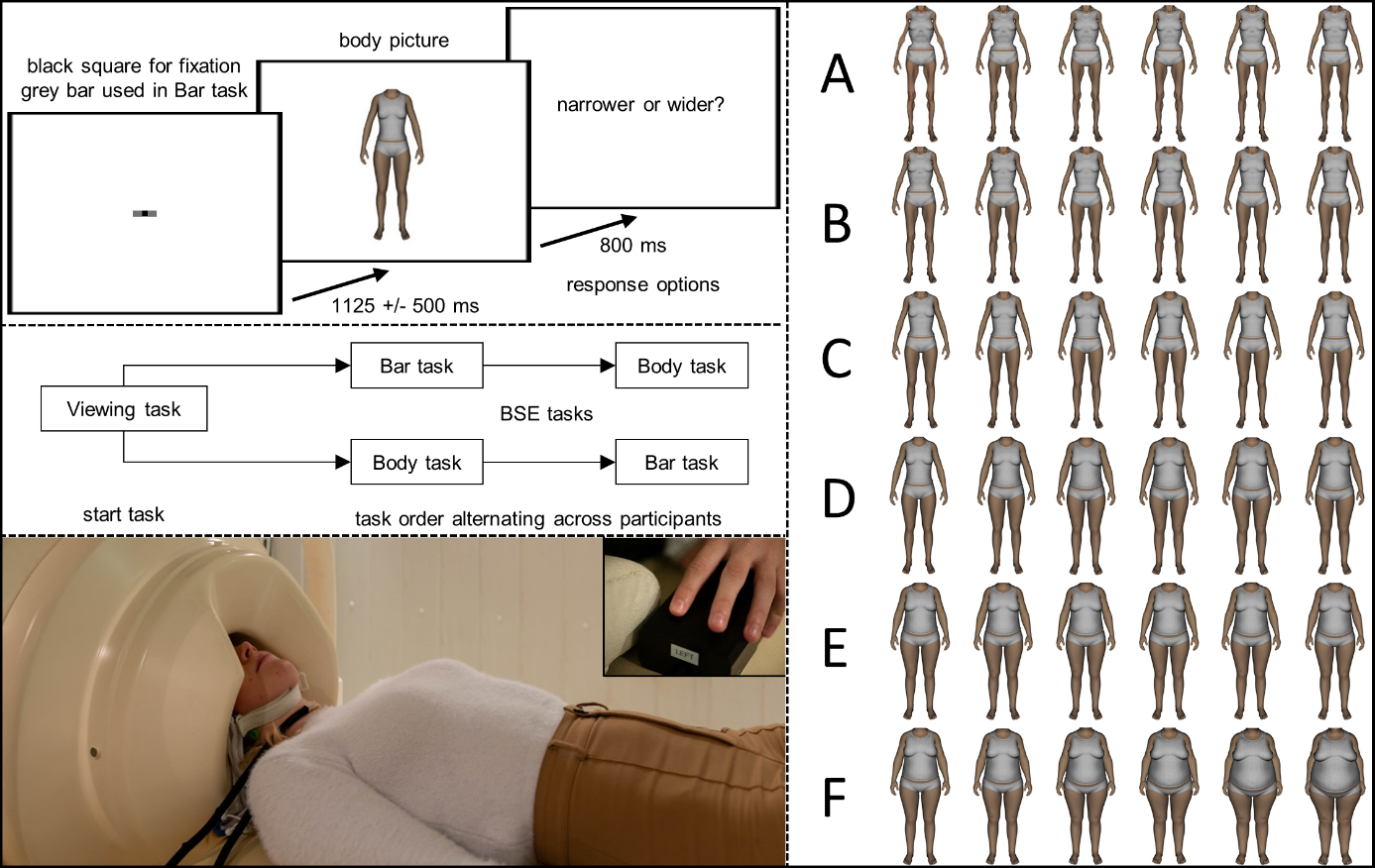
**Supplementary Figure 6 a: Depictive BSE Paradigm (from Romero et al., 2024)**

Top left: Procedure. Participants were asked to attentively view the body pictures. In the viewing task, no further instruction was given, and no response options were presented. In the BSE tasks, participants used the response options “narrower” or “wider” and responded via button press. In the bar task, participants compared the width of the waistline of the previously presented body picture to the width of the central grey bar. In the body task, participants compared the previously presented body picture to their own body. Presentation order of the BSE tasks alternated across participants. Bottom left: Subject during parallel EEG and MEG recording and response device. Right: Body pictures in categories A–F from top to bottom.

**Supplementary Figure 6 b: Depictive BSE Results**


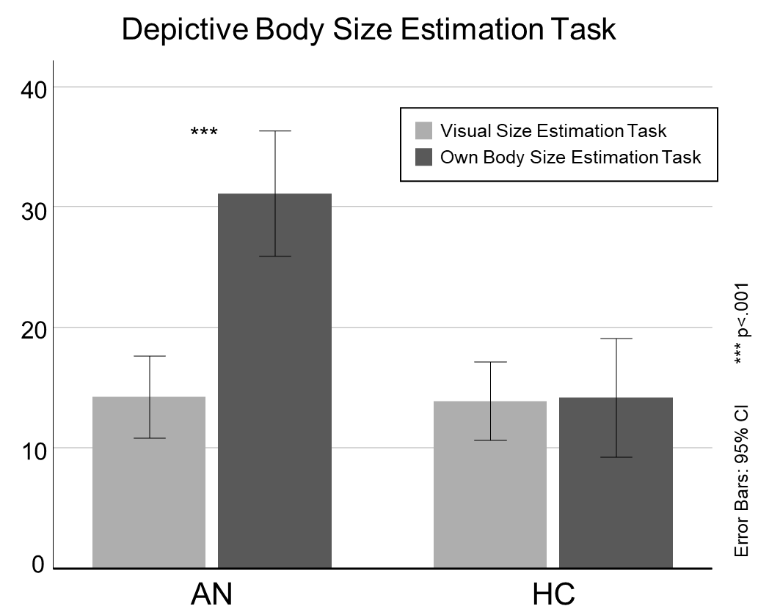


**Supplement 7: Distribution of F-values representing the main effect of group**

Supplementary figure 7 shows the distribution of F-values representing the main effect of group (which had been calculated for each neural source and each time point) prior to the application of cluster permutation tests correcting for multiple testing. The left panel shows that the MEG-based main effect of group, with reduced neural activity in the AN versus HC group in the inferior temporal cortex, was basically bilateral. However, in the left hemisphere, it did not reach the significance threshold of the highly conservative cluster permutation test. In contrast, the right panel shows that the EEG-based main effect of group in the PPC (mid-latency TOI) and inferior temporal cortex (late TOI) was lateralized to the left.

**Supplementary Figure 7**
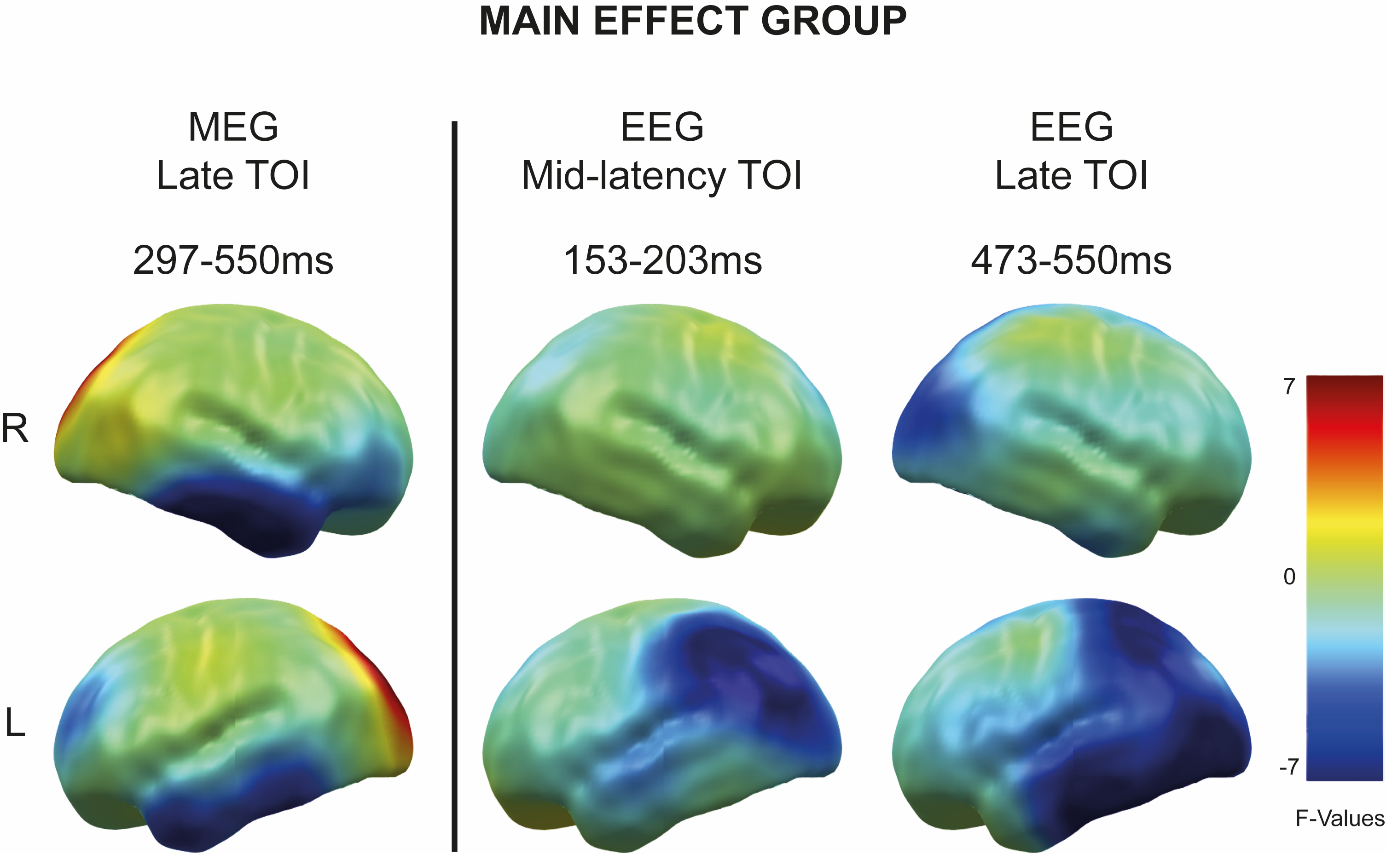


F-values in blue = neural activity AN < HC, F-values in red = neural activity AN > HC

Differences between EEG and MEG methods and the asymmetry of tactile processing are the most likely explanations for the observed hemispheric differences.

With respect to methodological aspects, differences between MEG- and EEG-based source localization may arise from their distinct sensitivities to neural currents and volume conduction properties. MEG is primarily sensitive to tangential sources and less affected by skull and scalp conductivity, whereas EEG captures both radial and tangential sources but is more strongly influenced by volume conduction and tissue inhomogeneities. Differences in sensor configuration and signal-to-noise ratio may further contribute to variability in localization. However, these modality-specific characteristics are unlikely to systematically produce hemispheric biases, as the underlying physical principles apply symmetrically. Apparent hemispheric differences may nevertheless arise under specific conditions, such as asymmetric head geometry, conductivity profiles, data quality, or interactions between source orientation and modality sensitivity (e.g., radial vs. tangential sources). Such factors may have contributed to the observed hemispheric differences.

Moreover, MEG- and EEG-based results converge in the late TOI and the left inferior temporal cortex, suggesting that the group effect is most robust in this region. At the same time, the neural oddball effect appears to be left-lateralised, as indicated by an earlier onset (EEG-based cluster in the early TOI, Figure 3A) and, at a descriptive level, a more pronounced/spatially distributed effect (higher F-values; Figure 3A) in the contralateral left hemisphere. Notably, regions showing neural differentiation between tactile stimuli overlapped with those in which AN patients exhibited reduced activity relative to HCs. Taken together, greater involvement of the contralateral left hemisphere in the present paradigm may contribute to the emergence of left-sided effects.
